# Phylogeography of Bivalve *Cyclina sinensis*: Testing the Historical Glaciations and Changjiang River Outflow Hypotheses in Northwestern Pacific

**DOI:** 10.1371/journal.pone.0049487

**Published:** 2012-11-07

**Authors:** Gang Ni, Qi Li, Lingfeng Kong, Xiaodong Zheng

**Affiliations:** The Key Laboratory of Mariculture, Ministry of Education, Ocean University of China, Qingdao, China; George Washington University, United States of America

## Abstract

**Background:**

The marginal seas of northwestern Pacific are characterized by unique topography and intricate hydrology. Two hypotheses have been proposed to explain genetic patterns of marine species inhabiting the region: the historical glaciations hypothesis suggests population genetic divergence between sea basins, whereas the Changjiang River outflow hypothesis suggests genetic break in line with the Changjiang Estuary. Here the phylogeography of bivalve *Cyclina sinensis* was investigated to test the validity of these two hypotheses for intertidal species in three marginal seas—the East China Sea (ECS), the South China Sea (SCS), and the Japan Sea (JPS).

**Methodology/Principal Findings:**

Fragments of two markers (mitochondrial COI and nuclear ITS-1) were sequenced for 335 individuals collected from 21 populations. Significant pairwise Φ_ST_ were observed between different marginal sea populations. Network analyses illustrated restricted distribution of haplogroups/sub-haplogroups to sea basins, with a narrow secondary contact zone between the ECS and SCS. Demographic expansion was inferred for ECS and SCS lineages using mismatch distributions, neutral tests, and extended Bayesian Skyline Plots. Based on a molecular clock method, the divergence times among COI lineages were estimated dating from the Pleistocene.

**Conclusions:**

The phylogeographical break revealed for *C. sinensis* populations is congruent with the historical isolation of sea basins rather than the putative Changjiang River outflow barrier. The large land bridges extending between seas during glaciation allowed accumulation of mutations and subsequently gave rise to deep divergent lineages. The low-dispersal capacity of the clam and coastal oceanography may facilitate the maintenance of the historical patterns as barriers shift. Our study supports the historical glaciations hypothesis for interpreting present-day phylogeographical patterns of *C. sinensis*, and highlights the importance of historical glaciations for generating genetic structure of marine coastal species especially those with low-dispersal abilities in northwestern Pacific.

## Introduction

**Figure 1:**
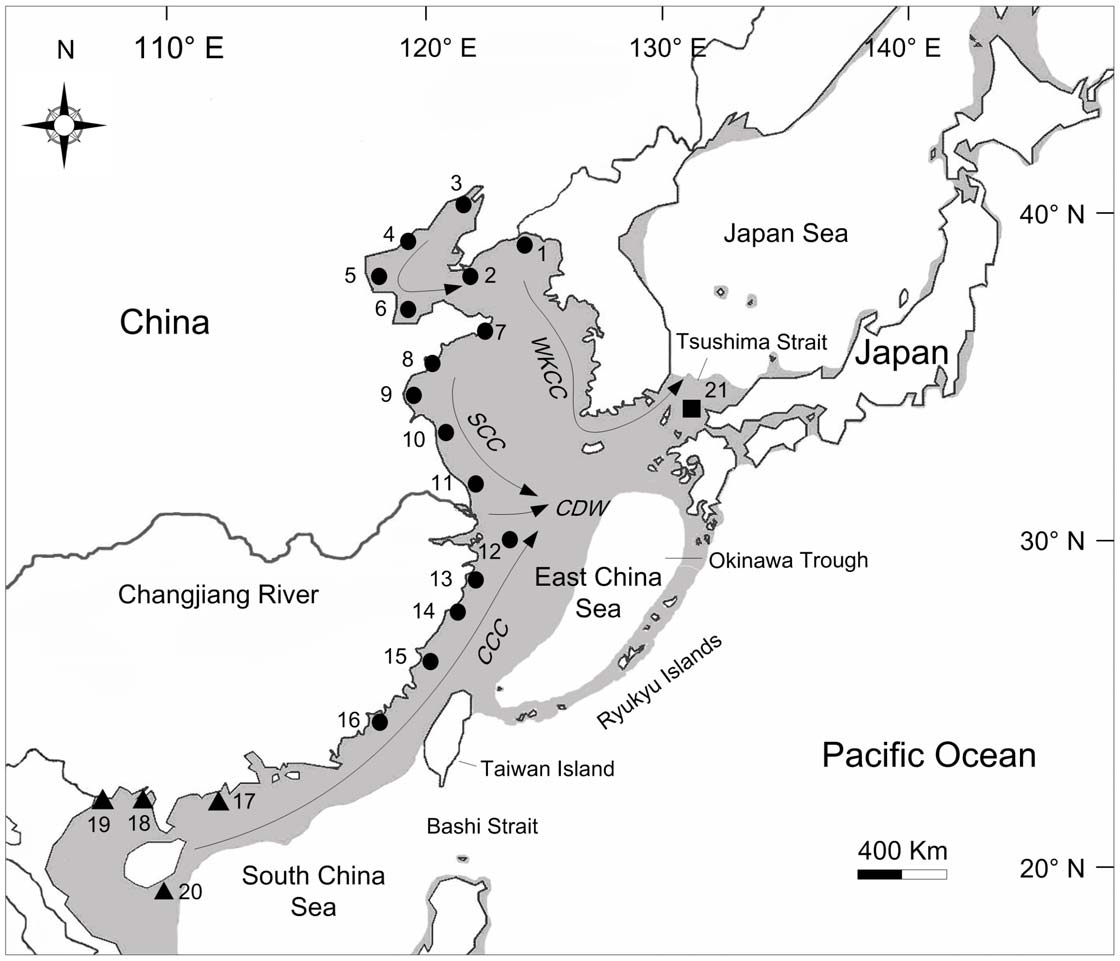
Map of East Asia showing the sampling sites of *Cyclina sinensis* and the coastal currents in summer. Populations are labelled with numbers as shown in Table 1. Different symbols are used to represent populations from three marginal seas: circles, East China Sea populations; triangles, South China Sea populations; square, Japan Sea population. Shaded sea areas indicate regions < 120 m depth that would have been exposed during periods of low sea level. WKCC, West Korea Coastal Current; SCC, Subei Coastal Current; CCC, China Coastal Current; CDW, Changjiang diluted water.

A series of marginal seas that has historically connected and isolated Asia from the Pacific Ocean contributes to the intricate geography of the northwestern Pacific [1]. When the last Pleistocene glaciers advanced, the sea level in the East China Sea (ECS) declined about 130–150 m than the present level while the South China Sea (SCS) was 100–120 m lower [2]. The ECS was reduced to an elongated trough (the Okinawa Trough) and the SCS became an enclosed inland sea connected to the Pacific through the Bashi Strait (Fig. 1). The ECS and SCS were thus isolated due to a large bridge extending from eastern China to Taiwan Island [3]. The Japan Sea (JPS) and ECS were also separated from each other when the Tsushima Strait was almost exposed (Fig. 1).

The historical separation of the sea basins was reported to have dramatically influenced the current genetic distribution of various marine species [4]–[7]. A general phylogeographical pattern resulting from the historical glaciation hypothesis is concluded [5]: three marginal seas had served as separate refugia and accumulated substantial genetic differentiation during glacial periods; two secondary contact zones were formed through postglacial expansion of previously isolated lineages at the adjacent region (ECS/JPS and ECS/SCS). The intertidal species are particularly expected to be impacted by sea level changes as they experienced a direct loss of habitat from ice scouring (moving sea ice ridges can scour the seabed in shallow waters) [8]. These species are likely to exhibit notable genetic differentiation between separate refugial populations, and maintain the signature of historical patterns over long time-scales as barriers shift [9].

However, the simple picture of genetic distribution drawn from climate changes may be obscured by other environmental factors. Oceanic currents operating during the pelagic phase theoretically facilitate the dispersal of larval and enhance high population connectivity [10], [11]. In summer, the Subei Coastal Current (SCC) brings low salinity water southward along the ECS coast, and the China Coastal Current (CCC) flows northward from the SCS into the ECS [12]. Meanwhile, the West Korea Coastal Current (WKCC) connects the ECS and JPS (Fig. 1). Another dominant factor is the Changjiang River (also known as the Yangtze River, Fig. 1) outflow, a putative physical barrier limiting gene flow of coastal species in the region [13]. As the third largest river in the world, the river pours into the ECS with an average annual discharge of 924 billion m^3^ [12]. The huge freshwater outflow named Changjiang diluted water (CDW) was reported to have profoundly influenced the hydrological and ecological features of the ECS [14], [15]. The CDW has been assumed as a major barrier for genetic arrangements of various marine species, including gastropod *Cellana toreuma* [13], bivalve *Cyclina sinensis* [16], and two varieties of *Sargassum* [17]. In fact, the Changjiang Estuary was assumed to be a biogeographical boundary for marine molluscan fauna a half-century ago [18]. However, Xu [19] pointed out that the boundary was not insurmountable for some bivalve species with broad temperature tolerance (such as *C. sinensis*), and they had the abilities to transgress the barrier and dispersed over a wide range. Thereby, the barrier effect of the Changjiang River outflow on gene flow of intertidal species (the Changjiang River outflow hypothesis) is still a controversial issue, and additional data are needed to appraise the influence cautiously.

The bivalve *C. sinensis*, widely distributed in East Asia, provides an ideal case with which to address the relative influence of historical glaciations and the Changjiang River outflow on the genetic structure of intertidal species. The two factors may affect population structure of *C. sinensis* on different temporal and spatial scales, resulting in different patterns with regard to group partitioning and location of secondary contact zones. This bivalve is considered a euryhaline species because it inhabits broad salinity ranges from muddy sand beaches in the intertidal zone to estuaries where freshwater enters sea [20]. Each female may release 30 000–40 000 oocytes at once during the main breeding season from July to September. The clam has relatively short planktonic larval duration (PLD), spending an average of 7 days in water column before settlement and metamorphoses [21]. Adult individuals of *C. sinensis* have home ranges no wider than 80 m [22]. These low-dispersal characteristics make *C. sinensis* particularly suitable to disentangle the complex interactions of various factors on genetic structure in marginal seas. Indeed, several studies have emerged with aims to reveal the genetic distribution of *C. sinensis* [16], [23]–[25]. Nevertheless, these studies are suffered from insufficient sampling with restricted spatial coverage that can lead to erroneous conclusions regarding patterns of differentiation, and contradictory results have been reported. Yao et al. [24] using random amplified polymorphic DNA found that populations from two sides of the Changjiang River showed close genetic relationship, while Zhao et al. [25] using amplified fragment length polymorphism revealed significant genetic differentiation in line with the river. This discordance may partially result from the application of genetic markers with different levels of resolution for genetic differences.

In the present study, we surveyed the phylogeographical structure of *C. sinensis* in three marginal seas (ECS, SCS, and JPS) of East Asia by intensive sampling with an emphasis on the coast of China. Based on the analysis of mitochondrial cytochrome oxidase I (COI) and nuclear ribosomal internal transcribed spacers 1 (ITS-1) markers variation, we specifically aimed at elucidating the detailed genetic patterns, and testing the validity of the historical glaciations and Changjiang River outflow hypotheses for the clam. By addressing these issues, we expect to gain better understanding of the genetic distribution as well as evolutionary mechanisms for marine coastal species in the northwestern Pacific.

## Materials and Methods

### Sample collection

All the specimens of wild *Cyclina sinensis* were collected between October 2005 and January 2010. We obtained 335 individuals from 21 localities representing the geographical distribution of this species in East Asia, with a range of 5–23 individuals per site (Fig. 1 and Table 1). The adductor muscle was incised from samples and stored in 95% ethanol immediately, except for specimens from five sites (Lvshun, Tianjin, Weifang, Xiamen, and Maoming) which were frozen at −30°C until DNA extraction. Total genomic DNA was isolated from muscle tissue by a modification of standard phenol-chloroform purification procedure described by Li et al. [26]. As *C. sinensis* is not an endangered or protected species and collections were only carried on from public access areas, no specific permits were required to collect this species from these locations/activities.

**Table 1:** Sampling information and diversity indices for 21 populations of *Cyclina sinensis*.

Sampling site	Abbr.	COI					ITS-1				
		*N_C_*	*n_C_*	*h_C_*	*π_C_*	*k_C_*	*N_I_*	*n_I_*	*h_I_*	*π_I_*	*k_I_*
**East China Sea**											
1 Dandong	DD	14	7	0.758	0.0024	1.512					
2 Lvshun	LS	18	4	0.634	0.0026	1.598					
3 Panjin	PJ	12	2	0.167	0.0003	0.174					
4 Qinhuangdao	QHD	20	5	0.716	0.0021	1.326					
5 Tianjin	TJ	18	8	0.778	0.0023	1.411					
6 Weifang	WF	19	6	0.743	0.0022	1.368					
7 Haiyang	HY	14	7	0.824	0.0029	1.790					
8 Jimo	JM	15	6	0.762	0.0023	1.411	8	8	1.000	0.0073	3.898
9 Lianyungang	LYG	15	8	0.838	0.0035	2.157	8	8	1.000	0.0058	3.078
10 Xiangshui	XS	11	6	0.855	0.0044	2.768					
11 Qidong	QD	13	3	0.295	0.0014	0.875	10	10	1.000	0.0129	6.810
12 Shengsi	SS	5	4	0.900	0.0035	2.245					
13 Zhoushan	ZS	10	4	0.778	0.0018	1.130	10	10	1.000	0.0121	6.391
14 Wenzhou	WZ	14	7	0.813	0.0027	1.706	7	6	0.952	0.0039	2.092
15 Xiapu	XP	23	7	0.783	0.0029	1.808	10	10	1.000	0.0087	4.617
16 Xiamen	XM	17	3	0.324	0.0007	0.431	9	7	0.944	0.0485	25.64
**South China Sea**											
17 Maoming	MM	17	1	0.000	0.0000	0.000	10	10	1.000	0.0105	5.236
18 Beihai	BH	17	2	0.118	0.0002	0.129	9	8	0.972	0.0082	4.053
19 Dongxing	DX	19	5	0.591	0.0011	0.695	7	6	0.952	0.0112	5.617
20 Sanya	SY	21	2	0.095	0.0002	0.107	10	10	1.000	0.0087	4.344
**Japan Sea**											
21 Fukuoka	FU	23	3	0.170	0.0030	0.174	12	10	0.970	0.0030	1.497

Number of individuals sampled per site (*N*), number of haplotype (*n*), haplotype diversity (*h*), nucleotide diversity (*π*), and mean number of pairwise differences (*k*) were shown for each population. Subscripts indicate variables for COI or ITS-1. Abbr, site abbreviation.

### Sequence acquisition

A fragment of the COI gene was amplified for all samples with the universal primers of Folmer et al. [27]. Each polymerase chain reaction (PCR) was carried out in 50- µL volumes containing 2 U Taq DNA polymerase (Takara Co.), about 100 ng template DNA, 0.25 µM of each primer, 0.2 mM dNTPs, 1×PCR buffer and 2 mM MgCl_2_. The PCR amplification was performed on a GeneAmp^®^ 9700 PCR System (Applied Biosystems) based on following conditions: initial denaturation at 94°C for 3 min, followed by 35 cycles of denaturation at 94°C for 1 min, annealing at 50°C for 1 min, and extension at 72°C for 1 min, and a final extension at 72°C for 5 min.

To confirm the COI results, the ITS-1 region was amplified for 110 individuals from 12 populations (Table 1) using the ITS1-a and ITS1-b primers described in Gaffney et al. [28]. Considering the intensive sampling and patterns revealed by COI in ECS, not all populations in ECS were sequenced for ITS gene (individuals in sites 1–7 and 12 were excluded). The PCR cocktails and conditions were as described above, except for the primer set and the annealing temperature (52°C for ITS-1). Amplification products were confirmed by 1.5% TBE agarose gel electrophoresis stained with ethidium bromide. The cleaned product was prepared for sequencing using the BigDye Terminator Cycle Sequencing Kit (ver. 3.1, Applied Biosystems) and sequenced on an ABI PRISM 3730 (Applied Biosystems) automatic sequencer. Multiple peaks of ITS-1 amplifications (suggesting the presence of more than one allele) were rare for this species as seen in some other organisms such as bivalves *Acar* [29] and velvet ant [30].

### Sequence variation

Sequences of COI and ITS-1 were assembled and edited by the DNASTAR software (DNASTAR, Inc.) separately, then aligned with CLUSTAL_X 1.81 using default settings [31], and finally rechecked by eye. The number of haplotypes for each locus was determined with the software program DnaSP v5 [32]. Sites with gaps in the ITS-1 sequences were considered as we thought haplotypes separated by an indel event to be different. All sequences of haplotypes were deposited in GenBank database (COI accession numbers: GU078392-GU078429, HM021146-HM021149; ITS-1 accession numbers: HQ232852-HQ232945). ARLEQUIN 3.5 software package [33] was applied to calculate molecular diversity indices such as haplotype diversity (*h*), nucleotide diversity (*π*), and mean number of pairwise differences (*k*). The evolutionary model that best fitted the two loci was determined with jModelTest [34] using the Akaike information criterion (AIC). HKY + I and HKY + G were chosen as the best-fit model for COI and ITS-1 loci respectively and used in the subsequent analyses.

### Phylogenetic analyses

Networks of haplotypes were constructed with 95% maximum parsimony threshold in TCS version 1.21 [35]. Gaps in ITS-1 haplotypes were treated as a fifth character state. While it is more desirable to treat indels as additional coded characters than as a fifth state, TCS does not currently support the inclusion of symbols in the data file. A fixed connection limit at 50 steps was used to ITS-1 haplotypes in cases of they were too distant for connection in a network.

To confirm the phylogenies among haplotypes, Bayesian inferences (BI) were performed in MrBayes version 3.1 [36]. The Markov-chain Monte Carlo search was run with four chains for 8 million (COI) and 20 million (ITS-1) generations with sampling frequency of 1/1000 trees. The gap information for ITS-1 sequences was encoded by the simple insertion/deletion coding [37], and the standard discrete model was used to this binary sequence data [38]. When the standard deviation of split frequencies was less than 0.01 at 3 million (COI) and 12 million (ITS-1) generations, parameter stationarity was achieved. Trees sampled prior to stationarity were discarded and then a 50% majority-rule consensus tree with branch lengths was constructed with the remaining trees. The net average genetic distance between major lineages was then calculated in MEGA 5 [39] using the Tamura-Nei model [40] (with gamma correction for ITS-1, G = 0.22), the closest model to the HKY in MEGA. To avoid introducing potential discrepancy to time estimates, we used he more stable COI marker to generate a relative time frame for lineage divergence based on COI divergence rates estimated for bivalve Arcidae (0.7% Myr^−1^) [41] and gastropod teguline (2.4% Myr^−1^) [42].

### Population structure analyses

Pairwise genetic divergences between populations were assessed using the fixation index Φ_ST_ as implemented in ARLEQUIN 3.5. Because the HKY model given by jModelTest is not available in ARLEQUIN, the Tamura-Nei model (with a gamma distribution for ITS-1) was used to correct the genetic distances. The significance of each pairwise comparison was tested with 10 000 random replicates, and a standard Bonferroni correction was applied for multiple tests [43]. An isolation by distance (IBD) analysis was conducted for all China Sea populations (sites 1–20, Table 1) using COI gene with Mantel tests, to examine the association between the genetic distance and geographical distance (log-transformed) using the IBDWS program [44]. Analyses were also performed for the ECS (sites 1–14, two hybrid populations 15 and 16 were excluded) and SCS (sites 17–20) populations separately. The significance of the correlation was tested using permutation methods (10 000 randomizations).

Hierarchical analyses of molecular variance (AMOVA, [45]) were used to estimate population structure in ARLEQUIN 3.5. According to the distribution of haplotypes and significant Φ_ST_ between different sea populations (see results), we tentatively partitioned populations into three groups consistent with sea basins. The significance of Φ-statistic analogs (Φ_CT_, Φ_SC_ and Φ_ST_) was evaluated with 10 000 random permutations. The feasibility of this pre-defined groupings were then examined using a spatial analysis of molecular variance as implemented in SAMOVA version 1.0 [46], which can cluster populations into user-defined number of groups (*K*, here 2–10) using a simulated annealing approach to maximize the variance (*F*_CT_) among those groups.

### Historical demography

After identifying lineages using phylogenetic methods, extended Bayesian Skyline Plots (EBSPs) were executed to examine population size changes of lineages in Beast v1.7 [47]. This approach using multiple loci can generate more accurate estimates of population size than the old method Bayesian Skyline Plot (BSP) using a single locus [48]. Analyses were conducted separately for each sea lineage incorporating both COI and ITS-1 information (ECS lineage: COI-a1, -a2 + ITS1-a; SCS lineage: COI-b + ITS1-b; JPS lineage: COI-c + ITS1-c). Substitution models, clock models, and trees were unlinked across markers; HKY + G model was used for both loci. The mitochondrial COI was utilized as the stable reference marker in the analysis considering the evolutionary processes of ITS-1 may be complicated by various mechanisms [49]. A fixed mean substitution rate of 0.775% change per million years (Myr^−1^) and a generation time of 1 year [50] were assigned to convert the parameters to actual time quantities. This substitution rate was obtained by averaging the COI divergence rates used above and then dividing by two. Analyses were run for 100 million generations and sampled every 1000 generations with the first 10 million generations discarded as burn-in. To assure the consistency of the results, each run was repeated for at least three times using different random number seeds. In all runs, the effective sample size values for the parameters of interest were over 300. The results were visualized and checked using Tracer 1.5 [51].

As a comparison to the EBSP, we also calculated Fu's *F_S_* [52] and Ramos-Onsins and Rozas' *R*_2_ [53], along with their statistical significance (1000 permutations) using the program DnaSP v5. In cases where expansion was evident based on either of those statistics, statistics in mismatch distributions that test for a departure from sudden population expansion (sum of squared deviation [SSD]) and Harpending's raggedness index [RI]) were calculated in ARLEQUIN 3.5 [54], [55]. The significance of SSD and RI was assessed by 1000 parametric bootstrap replicates, and mismatch frequency histograms were plotted in DnaSP v5.

## Results

### Sequence variation

**Figure 2:**
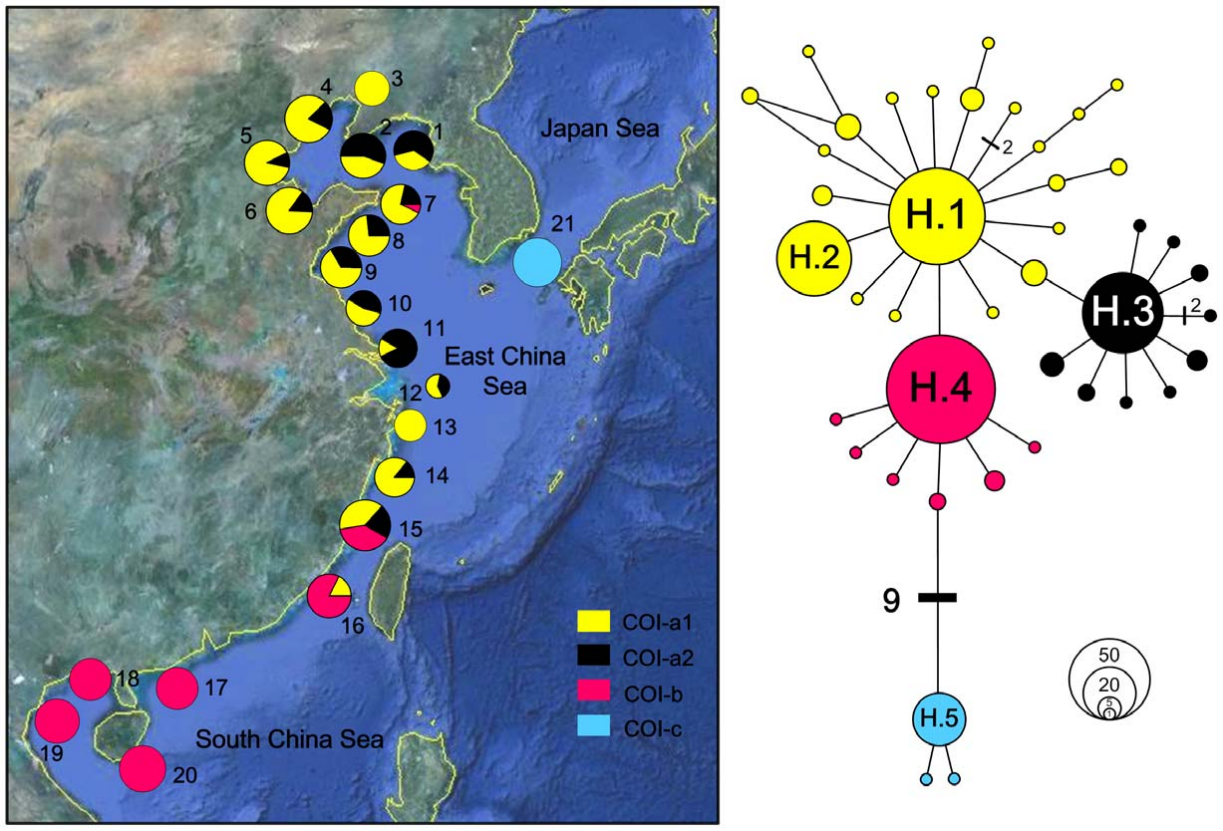
Network and spatial distribution of *Cyclina sinensis* COI haplotypes. For the network, two haplogroups representing populations in China and Japan were indicated. The three China sub-haplogroups and one Japan haplogroup are colour-coded separately: yellow, subgroup COI-a1; black, COI-a2; red, COI-b; blue, haplogroup COI-c. The sizes of circles are proportional to haplotype frequencies and are separated by one mutation step, unless otherwise indicated by bars with numbers. Five haplotypes with high frequencies are labelled with H.1 to H.5. The spatial distributions of the subgroups and group are shown in 21 populations (labelled with numbers as shown in Table 1) by pie diagrams.

An alignment of 624 bp COI gene fragment was analysed for 335 individuals from 21 localities. The COI data were characterized by low nucleotide diversity (the highest *π* = 0.0044 in Xiangshui, site 10) and sharp different haplotype diversity (ranging from 0 to 0.9; Table 1). A total of 42 different haplotypes were defined by 34 polymorphic sites with 32 transitions, two transversions and no indels. The haplotypes shared among populations were also the most abundant ones (for example, H.1, H.2, and H.3 in ECS populations and H.4 in six southernmost populations; Fig. 2). For Fukuoka (site 21) in Japan, three private haplotypes were found in 23 individuals and H.5 was the most common haplotype with 21 copies. The mean number of pairwise differences between individuals within localities ranged from 0 in Maoming (site 17) to 2.768 in Xiangshui (site 10).

The final ITS-1 alignment (Table S1) was 544 bp long included 46 parsimony informative sites, resulting in 94 unique haplotypes from 110 individuals. Overall, the ITS-1 locus exhibited considerably higher levels of haplotype diversity, nucleotide diversity and mean number of pairwise differences than COI (Table 1). Xiamen population (site 16) displayed the highest nucleotide diversity (*π* = 0.0485) as well as mean number of pairwise differences (*k* = 25.64).

### Phylogenetic analyses

Two haplogroups were formed in the COI network: one group comprised three haplotypes in JPS (COI-c, representing JPS COI lineage; Fig. 2), and the other comprised all haplotypes in ECS and SCS. This phylogeny was also confirmed by the Bayesian inference in which the similar topology was indicated (results not shown). The haplotypes in JPS were notably different from those in China Sea as at least 9 bp substitutions were required to connect them. Further resolution can subdivide the China haplogroup into three star-like subgroups (COI-a1, -a2, and -b): COI-a1 and a2 co-distributed in most ECS populations (so COI-a1 and -a2 were treated together in the following analyses representing the ECS COI lineage), while COI-b was geographically distinct in SCS populations (SCS COI lineage). Significant overlaps among them were observed in two populations Xiapu (site 15) and Xiamen (site 16) located at the adjacent area of the ECS and SCS (Fig. 2). The net average genetic distances (± SE) between COI lineages were ECS/SCS: 0.18% (0.17%); ECS/JPS: 1.80% (0.54%); SCS/JPS: 1.72% (0.54%). Based on the divergence rates of 0.7–2.4% Myr^−1^, the split times between the ECS and SCS COI lineages occurred about 74 000 to 254 000 years ago, while the divergence time for ECS/JPS and SCS/JPS dating from 750 000 to 2 570 000 and 720 000 to 2 460 000 years ago, respectively. All these dates fall into the range of Pleistocene period from 11 700 to 2 588 000 years ago [56].

**Figure 3:**
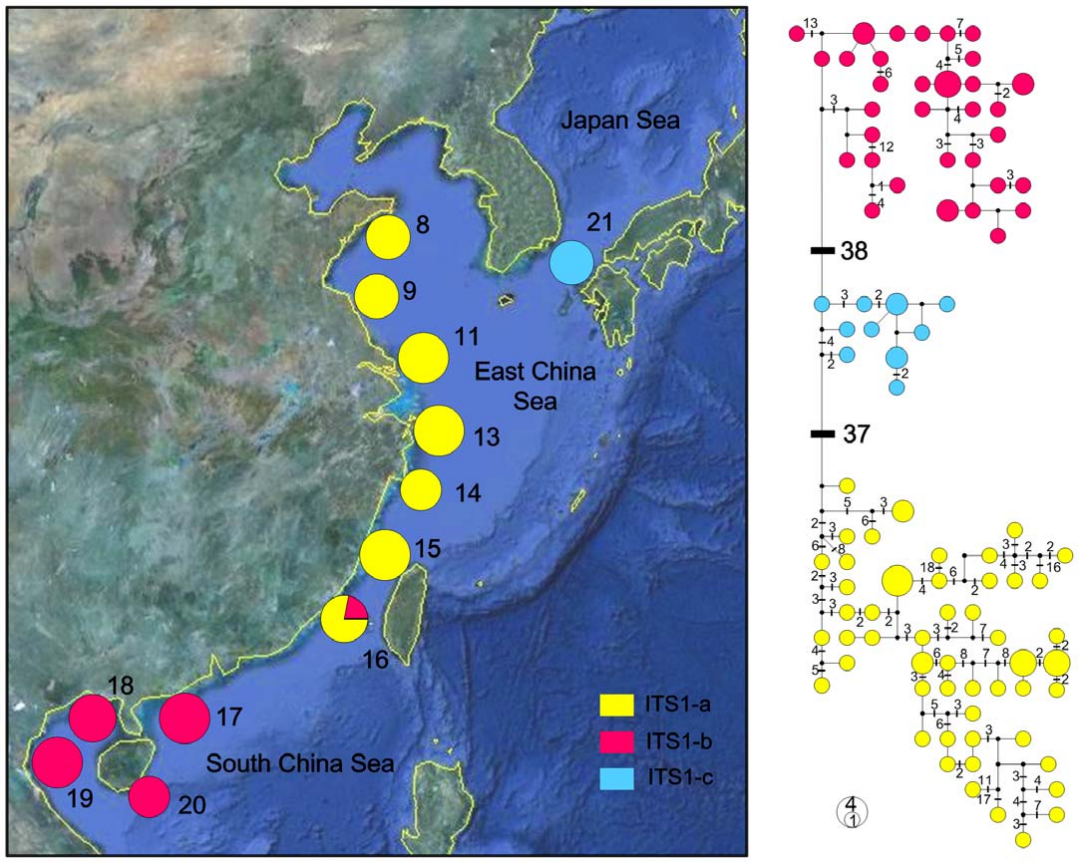
Network and spatial distribution of *Cyclina sinensis* ITS-1 haplotypes. Three haplogroups showed in the network are colour-coded separately: yellow, ITS1-a; red, ITS1-b; blue, ITS1-c. The sizes of circles are proportional to haplotype frequencies and are separated by one mutation step, unless otherwise indicated by bars with numbers. The small black dots indicate hypothetical missing haplotypes. The spatial distributions of three haplogroups are shown in populations (labelled with numbers as shown in Table 1) by pie diagrams.

**Figure 4:**
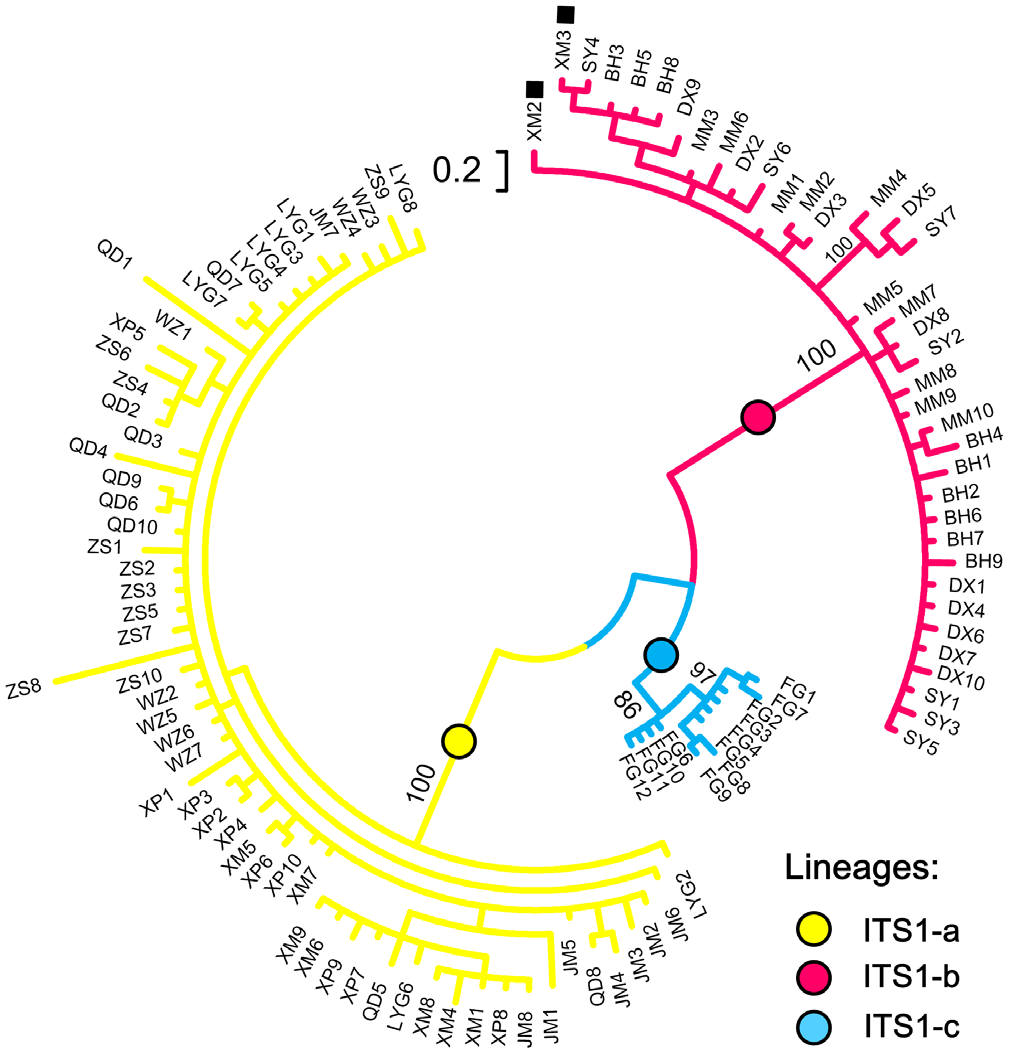
The Bayesian tree of ITS-1 haplotypes. Numbers above branches are Bayesian posterior probabilities for the main lineages. Support values less than 50% are not shown. Two individuals in Xiamen locality (site 16, ECS) observed in SCS lineage ITS1-b are indicated using black squares.

The ITS-1 network (Fig. 3) and Bayesian inference (Fig. 4) retrieved the same gross topology as three distinct haplogroups were shown (ITS1-a, -b, and -c). Each of them was found geographically restricted in one marginal sea, that is, ITS1-a in ECS, ITS1-b in SCS, and ITS1-c in JPS (Fig. 3). The net average genetic distances between ITS-1 lineages (± SE) were ECS/SCS: 8.51% (2.17%); ECS/JPS: 5.44% (1.64%); SCS/JPS: 4.48% (1.30%). Lineage overlap was only observed between ITS1-a and -b in one population (Xiamen, site 16).

### Population analyses

**Figure 5:**
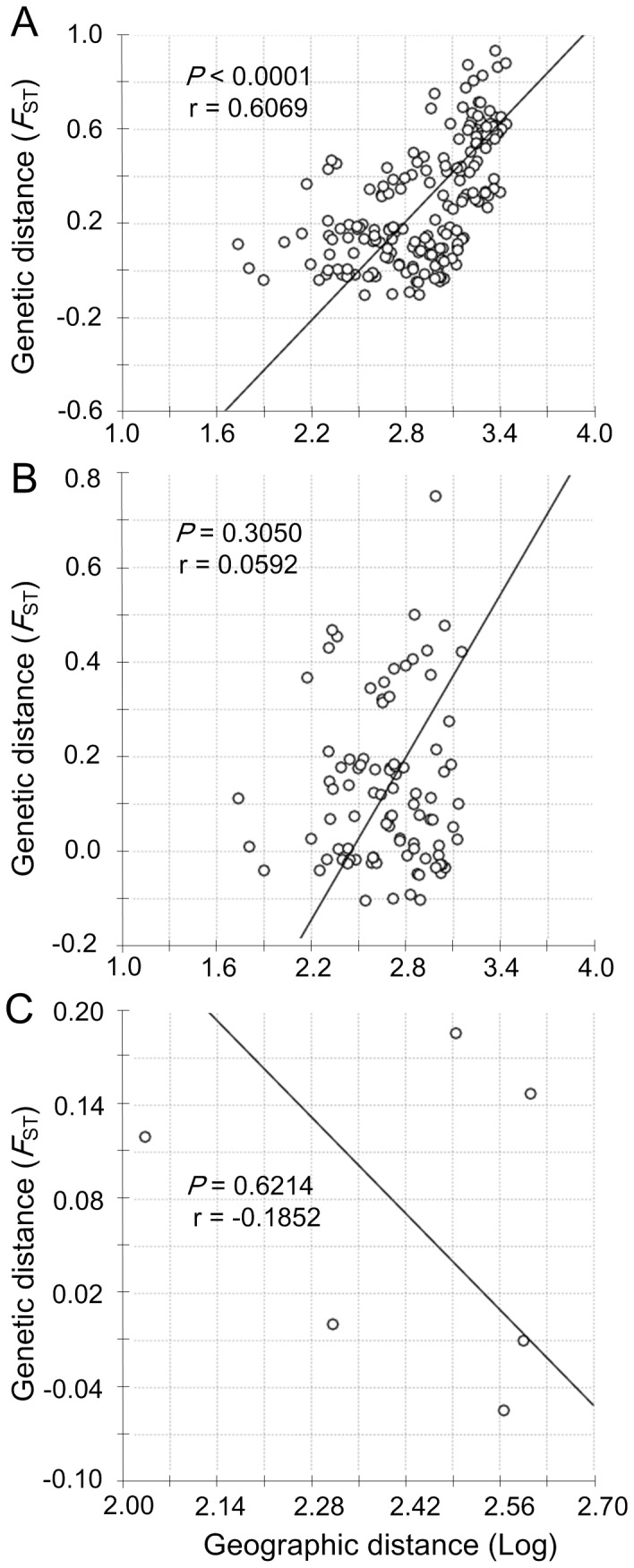
Isolation by distance plots for three grouping of *Cyclina sinensis*. Relationship between genetic distance and geographic distance (log-transformed) for (A) all China populations (sites 1–20); (B) the ECS populations (sites 1–14, two hybrid populations 15 and 16 were excluded); (C) the SCS populations (sites 17–20).

Nearly all pairwise Φ_ST_ values between populations of different seas were high and significant after Bonferroni corrections for both molecular markers (Table S2 and S3). Significant values were also observed for populations within ECS. Xiamen population (site 16) dominated by subgroup COI-b was remarkably different from other ECS populations for COI analysis (Φ_ST_ values range from 0.219, *P* = 0.006, to 0.850, *P* < 0.001; Table S2). Besides this site, significant values were observed for other 12 comparisons based on COI data. For the ITS-1, Xiamen population was found significantly different from ZS population (Φ_ST_ = 0.187, *P* < 0.001; Table S3). The IBD analysis revealed significant correlation between genetic and geographic distances for all China populations (*P* < 0.0001, r = 0.6069; Fig. 5A). However, when the ECS and SCS populations were analyzed separately, no significant correlation was observed for either group [ECS: *P* = 0.3050, r = 0.0592 (Fig. 5B); SCS: *P* = 0.6214, r = −0.1852 (Fig. 5C)].

Of each SAMOVA grouping strategy, the grouping for which the *F*_CT_ value was highest was selected as the best grouping method. The analysis based on ITS-1 marker showed the highest *F*_CT_ appeared for three groups of populations (*F*_CT_ = 0.876), which was highly concordant to the pre–defined grouping in AMOVA. For the COI data, the populations were primarily divided into two clusters (China group and Japan group, *F*_CT_ = 0.856). When only China group considered, the highest variation among group appeared for two groups (*F*_CT_ = 0.440) mainly coinciding with the ECS and SCS (except for Xiamen population in the ECS assigned to SCS). These results in SAMOVA validated the pre-defined groupings in AMOVA based on sea basins. Hierarchical analyses of AMOVA groupings indicated that most of the genetic variation could be attributed to variation among groups (COI: 73.3%; ITS-1: 87.6%), followed by variance within populations (COI: 21.0%; ITS-1: 11.4%). Only 5.7% for COI and 1.0% for ITS-1 were due to variation among populations within groups, but the variance components at all levels were statistically significant (all *P* < 0.05, Table 2).

**Table 2:** Results from analysis of molecular variance (AMOVA) of population structure in *Cyclina sinensis*.

Marker/Grouping	Source of variation	d.f.	Φ-Statistics	% of variation	*P* value
COI	Among groups	2	Φ_CT_ = 0.732	73.3	**0.0025**
(Group I: 1–16; II: 17–20; III: 21)*	Among populations within groups	18	Φ_SC_ = 0.212	5.7	**< 0.0001**
	Within populations	314	Φ_ST_ = 0.790	21.0	**< 0.0001**
					
ITS-1	Among groups	2	Φ_CT_ = 0.876	87.6	**0.0004**
(Group I: 8, 9, 11, 13–16; II: 17–20; III: 21)*	Among populations within groups	9	Φ_SC_ = 0.084	1.0	**0.0140**
	Within populations	90	Φ_ST_ = 0.886	11.4	**< 0.0001**

Significant *P* values are bolded. * Populations were assigned to three groups consistent with sea basins. See Table 1 for site number.

### Historical demography

**Figure 6:**
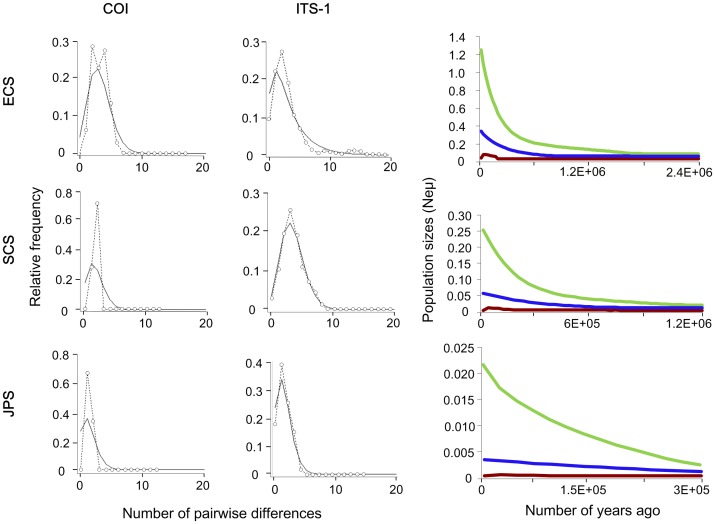
Mismatch distributions and extended Bayesian skyline plots (EBSPs) for three lineages. In mismatch distributions, dotted lines with circles represent the observed frequency of pairwise differences, whereas the solid lines show the expected values under the sudden population expansion model. The EBSPs show the demographic trends for three lineages as expressed in years. Blue lines represent median population estimates; green and brown lines denote upper and lower 95% confidence intervals.

Reconstruction of population sizes through time with the EBSP approach indicated two lineages (ECS and SCS lineages) have experienced significant population size changes (Fig. 6). The 95% highest posterior distribution (HPD) of the estimated number of size-change steps for these lineages did not include zero (1–2 and 1–3, respectively). To the contrary, the population size of JPS lineage maintained stable throughout the evolutionary history (number of size-change steps 0–2). After a prolonged stable period, the population sizes for ECS and SCS lineages both increased from about 600 000 years ago. The ECS lineage had the longest evolutionary history beginning about 2.4 Myr ago, while the history for the SCS and JPS lineages was relatively short, from about 1 200 000 and 300 000 years ago, respectively.

The Fu' *F*_S_ and the Ramos-Onsins and Rozas' *R*_2_ (Table 3) showed all lineages except for ITS-c had significant values (*P* < 0.05), implying a demographic expansion event under the neutral model. Mismatch distribution analyses for all lineages (except for ECS COI lineage) uniformly displayed unimodal distributions (Fig. 6), indicating that each of them has experienced a demographic expansion. The RI and SSD of ITS1-a, -b, and COI-c coincided with the null hypothesis of sudden expansion model with nonsignificant values (*P* > 0.05, Table 3).

**Table 3:** Estimates of neutral tests (Fu's *F_S_* and Ramos-Onsins and Rozas' *R*_2_) for population expansion of each lineage and the mismatch distribution parameters RI and SSD.

Sea basin/Lineage		Fu' *F*_S_	*P*	*R*_2_	*P*	RI	*P*	SSD	*P*
ECS	COI-a1, a2	−51.01	**<0.001**	0.042	**<0.001**	0.087	0.013	0.021	0.014
	ITS1-a	−26.98	**<0.001**	0.045	**0.004**	0.002	**0.927**	0.002	**0.723**
SCS	COI-b	−6.316	**<0.001**	0.058	**<0.001**	0.776	0.041	0.213	0.010
	ITS1-b	−22.89	**<0.001**	0.071	0.078	0.008	**0.761**	0.006	**0.331**
JPS	COI-c	–*	–	0.236	**<0.001**	0.667	**0.620**	0.164	**0.170**
	ITS1-c	−2.264	0.069	0.132	0.170	–	–	–	–

RI  =  raggedness index, SSD  =  sum of squared deviations. Nonsignificant values for RI and SSD (*P* > 0.05) and significant values for *F*_S_ and *R*_2_ (*P* < 0.05) are bolded. *No *F*_S_ value available as only three sequences in COI-c.

## Discussion

### Pleistocene-driven phylogeographical patterns in East Asia

In the present study, we applied mitochondrial and nuclear sequences to examine the genetic diversity and phylogeographical structure of *Cyclina sinensis* in three marginal seas of East Asia. The network relationships and BI for both markers revealed genetically divergent haplogroups/subgroups displaying clear geographical distribution in accordance with the sea basins: COI-a1, -a2 and ITS1-a in the ECS; COI-b and ITS1-b in the SCS; and COI-c and ITS1-c in the JPS (Fig. 2 and 3). Clear population structure was demonstrated among three seas as almost all pairwise Φ_ST_ values between different sea populations were high and significant. These results together can provide confident evidences that the present-day phylogeographical pattern of *C. sinensis* was most closely related with the historical isolation of marginal seas. No phylogeny distribution or population structure was found in line with the Changjiang River discharge. The optimal grouping method suggested by SAMOVA for each marker also showed that populations were mainly grouped according to the sea basins they located rather than the Changjiang River. The AMOVA analysis revealed that variation among groups contributed to most of the genetic variation, indicating the substantial genetic differentiation among sea basins.

The sea level falls occurring throughout the Pleistocene resulted in the isolation of marginal seas, which might serve as vicariant barriers for the clam and give rise to divergent lineages. The similar pattern was also observed for other bivalves in the region [7], [57], as well as for marine fishes [4], [6] and mitten crab [5], all of which uniformly suggested the separation of marginal seas shaping the basic patterns of genetic diversity and distribution in northwestern Pacific. Worldwide, in marine environments, historical glaciation is proved to be the most effective force in generating intraspecific genetic splits [58], such as in Indo-West Pacific [59], Atlantic and Mediterranean basins [60], [61], as well as northwestern Atlantic region [62]. Cautiously, as just one JPS population was included in this study, only if more sample sites are sampled can detailed population structure and evolutionary history be revealed in JPS.

As a comparison, obvious genetic breaks were infrequently reported to be resulted from freshwater outflow (except for the Amazon-Orinoco outflow in South America [63]), perhaps mainly due to potential historical changes of estuary, variable river discharges, and hidden passageways for gene flow in intricate ocean hydrology. In this study, the barrier effect of the Changjiang River outflow was not validated for *C. sinensis*. This euryhaline species may have the ability to fit the significantly decreased salinity environment around the Changjiang Estuary and maintain gene exchange between populations of both sides. For other marine molluscs in the region, no significant genetic divergence has been reported in line with the outflow (e.g. *Mactra veneriformis* [64], *Rapana venosa* [65], and *Tegillarca granosa* [66]) except for a rocky intertidal species *Cellana toreuma* [12]. Thus the river seems to affect some but not all molluscan species depending on differentiation in biological characteristics (such as PLD and salinity tolerance) and/or habitat specificity (exposed or sheltered shores). Note, as the river may influence genetic structure in a more recent historical scale, the conclusion here will be better supported by evidence from other markers with higher mutation rates (such as microsatellites) in future study.

### Different levels of genetic diversity and possible explanations

The mitochondrial COI and nuclear ITS-1 displayed significantly different levels of genetic diversity which are often seen in phylogeographical studies with multiple markers [67], [68]. Generally, animal mitochondrial DNA accumulates nucleotide substitutions severalfold faster than does nuclear DNA (see review by Avise [69]). However, surprisingly, the ITS-1 marker here displayed much higher level genetic variations than COI, and quite many steps were required to connect the three lineages. Although there is not yet a definitive explanation for this observation, several hypotheses have been suspected. A sex-biased gene flow for this dioecious diploid clam [70] could be initially assumed for the patterns as a consequence of greater dispersal of females than males. Since adult individuals of *C. sinensis* were proved with limited dispersal ability [22], the dispersal of the clam was mainly depended on passive dispersal via currents during PLD. However, considering both male and female larvae have the same opportunities to disperse, a sex-biased gene flow seems not be valid for the results. A second hypothesis may be mitochondrial selective sweeps in the SCS populations as an excess of low frequency polymorphisms was observed (Fig. 2). Because no recombination of mitochondrial genomes occurs, genetic hitch-hiking (the process of neutral alleles increase in frequency by virtue of being linked to a gene that is favoured by natural selection) has the potential to induce a strong selective sweep [71], causing the remaining set of haplotypes to deviate from predictions based on neutrality [72]. This hypothesis is supported by the significant negative values in Fu's *F*_S_ and Ramos-Onsins and Rozas' *R*_2_ neutral tests (both *P* < 0.001, Table 3). Another possible explanation is the non-coding ITS regions have fast evolutionary rate which can rapidly accumulate substitutions due to the weak pressure of selection [73]. Additionally, the speed and direction of concerted evolution of ITS is proved to be unpredictable and is not accordant across different descendant lineages [49], [74]. Therefore, although no overall mutation rates are calculated for COI and ITS-1 of bivalves, ITS lineages have great potential to go through different directional evolution and then show deeper genetic divergence than COI over long-term evolutionary time scale. This unusual phenomenon has also been reported in several studies including spider mites [75], freshwater sponges [76], and coral reef species [77], [78]. Finally, the ITS-1 belonging to the ribosomal multigene family is formed by hundreds to thousands of tandem copies usually located in separate chromosomes [49]. Due to much larger effective population size, the ITS-1 has great potential to harbour higher allelic variation and deep phylogeny than the maternally inherited and effectively haploid mitochondrial COI.

### Historical demography and post-glaciation dispersal

When sea level rose as glaciers melted, the survived intertidal species might experience demographic expansion and occupy the new habitat quickly [69]. Significant population size changes have been illustrated for the ECS and SCS lineages using coalescent-based approach EBSP, both of which started from 600 000 years ago in the middle Pleistocene. The expansion was corroborated by the results of mismatch distribution and neutrality tests for lineages of each marker (Table 3). However, these two lineages carried different genetic signatures of evolutionary history within marginal seas: the existence of several abundant haplotypes (H.1–H.3) and the relatively high haplotype diversity indicated that the ECS was the intensive region of genetic diversity going through long-term accumulation of mutations, while the history for the SCS might be much shorter. No significant expansion had been detected for the JPS lineage either by the EBSP analysis or the corresponding neutral tests. This may be due to the relative stable environmental changes in the JPS [5] or insufficient sampling of JPS populations in this study.

Postglacial range expansions might bring formerly isolated lineages into secondary contact at suture zones [79], [80]. In discordance with the two suture zones (ECS/JPS and ECS/SCS) revealed for marine fish [4] and mitten crab [5], secondary contact for *C. sinensis* was only observed between ECS and SCS lineages in a narrow geographic range from Xiapu (site 15) to Xiamen (site 16). No significant relationship was found between genetic and geographic distances for either ECS or SCS populations, indicating a non-IBD model of gene exchange within each sea. The IBD pattern observed for all China samples was mainly due to genetically divergent populations from either side of the suture zone were included. Although the CCC connects the ECS and SCS with a common velocity of 20 cm/s in summer [13] and the WKCC entered into the JPS, gene flow promoted by these currents among the three seas seems limited. This is most likely attributable to the low-dispersal ability of the clam and the existence of small but persistent reciprocal flow and rotating flow along coastal areas [81], which impede the exposure of larvae to currents and their ability to be transported effectively. Alternatively, there may be unseen selective gradients (such as temperature and salinity) maintaining spatial segregation of the lineages which need further confirmation.

## Conclusions

Our results supported the historical glaciations hypothesis for shaping the present-day phylogeographical structure of *Cyclina sinensis* in East Asia. No significant barrier effect of the Changjiang River outflow was revealed in the study. Detailed comparative investigations on other intertidal species are still needed to test and complement the perspective. Empirical studies in the future, focusing on the barrier effect of the Changjiang River discharge, should be cautiously carried out by sufficient sampling around the estuary and proper analyses. The present study contributes to the better understanding of historical processes of coastal species and disentangles complex interactions of various factors on generating biodiversity in the northwestern Pacific.

## Supporting Information

Table S1 **The final alignment of 110 ITS-1 sequences.**
(NEX)

Table S2 **Pairwise Φ_ST_ based on COI (below diagonal) and associated *P* values (above diagonal) among the 21 populations (see Table 1 for abbreviations).** Values in bold indicate significant *P* values after Bonferroni correction (n = 1000, *P* < 0.05).
(DOC)

Table S3 **Pairwise Φ_ST_ based on ITS-1 (below diagonal) and associated *P* values (above diagonal) among the 12 populations (see Table 1 for abbreviations).** Values in bold indicate significant *P* values after Bonferroni correction (n = 1000, *P* < 0.05).
(DOC)
