# Supplementary material for: Phylogeography of Bivalve Cyclina sinensis: Testing the Historical Glaciations and Changjiang River Outflow Hypotheses in Northwestern Pacific
Source: PLoS One. 2012 Nov 7;7(11):e49487. doi: 10.1371/journal.pone.0049487 (PMC3492281; doi:10.1371/journal.pone.0049487)
Supplement: Table S2 — Pairwise ΦST based on COI (below diagonal) and associated P values (above diagonal) among the 21 populations (see Table 1 for abbreviations). Values in bold indicate significant P values after Bonferroni correction (n = 1000, P < 0.05). (DOC) [file pone.0049487.s002.doc]

**Table S2.** Pairwise Φ_ST_ based on COI (below diagonal) and associated *P* values (above diagonal) among the 21 populations (see Table 1 for abbreviations). Values in bold indicate significant *P* values after Bonferroni correction (n = 1000, *P* < 0.05).

| Sites |  | ECS | | | | | | | | | | | | | | | |  | SCS | | | |  | JPS |
| --- | --- | --- | --- | --- | --- | --- | --- | --- | --- | --- | --- | --- | --- | --- | --- | --- | --- | --- | --- | --- | --- | --- | --- | --- |
|  |  | DD | LS | PJ | QHD | TJ | WF | HY | JM | LYG | XS | QD | SS | ZS | WZ | XP | XM |  | MM | BH | DX | SY |  | FU |
| DD |  | — | 0.344 | **0.001** | 0.006 | **0.001** | 0.002 | 0.007 | 0.044 | 0.018 | 0.976 | 0.391 | 0.167 | **< 0.001** | 0.003 | **0.001** | **< 0.001** |  | **< 0.001** | **< 0.001** | **< 0.001** | **< 0.001** |  | **< 0.001** |
| LS |  | -0.003 | — | 0.182 | 0.053 | 0.038 | 0.016 | 0.049 | 0.394 | 0.044 | 0.893 | 0.132 | 0.369 | 0.017 | 0.025 | 0.014 | **< 0.001** |  | **< 0.001** | **< 0.001** | **< 0.001** | **< 0.001** |  | **< 0.001** |
| PJ |  | 0.467 | 0.054 | — | 0.998 | 0.996 | 0.999 | 0.999 | 0.494 | 0.575 | 0.998 | **< 0.001** | 0.910 | 0.020 | 0.988 | 0.999 | **< 0.001** |  | **< 0.001** | **< 0.001** | **< 0.001** | **< 0.001** |  | **< 0.001** |
| QHD |  | 0.326 | 0.130 | -0.112 | — | 0.631 | 0.556 | 0.924 | 0.352 | 0.738 | 0.890 | **< 0.001** | 0.594 | 0.192 | 0.591 | 0.070 | **< 0.001** |  | **< 0.001** | **< 0.001** | **< 0.001** | **< 0.001** |  | **< 0.001** |
| TJ |  | 0.380 | 0.147 | -0.131 | -0.031 | — | 0.547 | 0.928 | 0.241 | 0.393 | 0.917 | **< 0.001** | 0.476 | 0.069 | 0.533 | 0.143 | **< 0.001** |  | **< 0.001** | **< 0.001** | **< 0.001** | **< 0.001** |  | **< 0.001** |
| WF |  | 0.390 | 0.209 | -0.114 | -0.032 | -0.026 | — | 0.791 | 0.166 | 0.653 | 0.470 | **< 0.001** | 0.462 | 0.230 | 0.643 | 0.026 | **< 0.001** |  | **< 0.001** | **< 0.001** | **< 0.001** | **< 0.001** |  | **< 0.001** |
| HY |  | 0.317 | 0.144 | -0.207 | -0.061 | -0.060 | -0.048 | — | 0.393 | 0.666 | 0.568 | **< 0.001** | 0.657 | 0.396 | 0.837 | 0.117 | **< 0.001** |  | **< 0.001** | **< 0.001** | **< 0.001** | **< 0.001** |  | **< 0.001** |
| JM |  | 0.178 | -0.009 | -0.029 | -0.002 | 0.020 | 0.053 | -0.010 | — | 0.362 | 0.980 | 0.005 | 0.739 | 0.043 | 0.168 | 0.113 | **< 0.001** |  | **< 0.001** | **< 0.001** | **< 0.001** | **< 0.001** |  | **< 0.001** |
| LYG |  | 0.216 | 0.146 | -0.032 | -0.047 | 0.001 | -0.038 | -0.039 | 0.005 | — | 0.317 | 0.002 | 0.835 | 0.258 | 0.706 | 0.007 | **< 0.001** |  | **< 0.001** | **< 0.001** | **< 0.001** | **< 0.001** |  | **< 0.001** |
| XS |  | -0.255 | -0.161 | -0.521 | -0.140 | -0.177 | -0.019 | -0.049 | -0.280 | 0.028 | — | 0.733 | 0.686 | 0.569 | 0.488 | 0.333 | 0.003 |  | **< 0.001** | **< 0.001** | **< 0.001** | **< 0.001** |  | **< 0.001** |
| QD |  | -0.015 | 0.102 | 0.666 | 0.486 | 0.533 | 0.542 | 0.470 | 0.351 | 0.361 | -0.129 | — | 0.021 | **< 0.001** | **< 0.001** | **< 0.001** | **< 0.001** |  | **< 0.001** | **< 0.001** | **< 0.001** | **< 0.001** |  | **< 0.001** |
| SS |  | 0.111 | -0.003 | -0.155 | -0.089 | -0.045 | -0.054 | -0.075 | -0.114 | -0.127 | -0.167 | 0.318 | — | 0.174 | 0.452 | 0.183 | **< 0.001** |  | **< 0.001** | **< 0.001** | **< 0.001** | **< 0.001** |  | **< 0.001** |
| ZS |  | 0.538 | 0.273 | 0.189 | 0.053 | 0.108 | 0.036 | -0.004 | 0.193 | 0.038 | -0.056 | 0.676 | 0.113 | — | 0.572 | 0.079 | **< 0.001** |  | **< 0.001** | **< 0.001** | **< 0.001** | **< 0.001** |  | **< 0.001** |
| WZ |  | 0.382 | 0.194 | -0.101 | -0.036 | -0.022 | -0.038 | -0.060 | 0.055 | -0.049 | -0.023 | 0.524 | -0.035 | -0.040 | — | 0.036 | **< 0.001** |  | **< 0.001** | **< 0.001** | **< 0.001** | **< 0.001** |  | **< 0.001** |
| XP |  | 0.338 | 0.183 | -0.193 | 0.079 | 0.040 | 0.121 | 0.062 | 0.062 | 0.202 | 0.015 | 0.466 | 0.084 | 0.095 | 0.117 | — | 0.006 |  | **< 0.001** | **< 0.001** | **< 0.001** | **< 0.001** |  | **< 0.001** |
| XM |  | 0.765 | 0.583 | 0.692 | 0.528 | 0.513 | 0.532 | 0.467 | 0.582 | 0.548 | 0.378 | 0.850 | 0.654 | 0.621 | 0.522 | 0.219 | — |  | 0.228 | 0.126 | 0.025 | 0.044 |  | **< 0.001** |
| MM |  | 0.824 | 0.659 | 0.953 | 0.633 | 0.631 | 0.637 | 0.582 | 0.685 | 0.630 | 0.475 | 0.896 | 0.770 | 0.772 | 0.630 | 0.330 | 0.094 |  | — | 0.999 | 0.997 | 0.999 |  | **< 0.001** |
| BH |  | 0.810 | 0.646 | 0.901 | 0.627 | 0.626 | 0.634 | 0.576 | 0.672 | 0.622 | 0.463 | 0.884 | 0.751 | 0.760 | 0.626 | 0.318 | 0.117 |  | 0.000 | — | 0.995 | 0.444 |  | **< 0.001** |
| DX |  | 0.780 | 0.630 | 0.646 | 0.582 | 0.572 | 0.585 | 0.534 | 0.623 | 0.606 | 0.469 | 0.850 | 0.680 | 0.648 | 0.583 | 0.316 | 0.017 |  | -0.099 | -0.085 | — | 0.873 |  | **< 0.001** |
| SY |  | 0.836 | .0680 | 0.907 | 0.650 | 0.646 | 0.655 | 0.605 | 0.702 | 0.653 | 0.512 | 0.901 | 0.787 | 0.776 | 0.650 | 0.352 | 0.102 |  | -0.010 | 0.001 | -0.031 | — |  | **< 0.001** |
| FU |  | 0.995 | 0.993 | 0.999 | 0.994 | 0.995 | 0.993 | 0.993 | 0.995 | 0.991 | 0.987 | 0.997 | 0.995 | 0.998 | 0.994 | 0.991 | 0.998 |  | 0.999 | 0.999 | 0.997 | 0.999 |  | — |
