# Supplementary material for: Phylogeography of Bivalve Cyclina sinensis: Testing the Historical Glaciations and Changjiang River Outflow Hypotheses in Northwestern Pacific
Source: PLoS One. 2012 Nov 7;7(11):e49487. doi: 10.1371/journal.pone.0049487 (PMC3492281; doi:10.1371/journal.pone.0049487)
Supplement: Table S3 — Pairwise ΦST based on ITS-1 (below diagonal) and associated P values (above diagonal) among the 12 populations (see Table 1 for abbreviations). Values in bold indicate significant P values after Bonferroni correction (n = 1000, P < 0.05). (DOC) [file pone.0049487.s003.doc]

**Table S3.** Pairwise Φ_ST_ based on ITS-1 (below diagonal) and associated *P* values (above diagonal) among the 12 populations (see Table 1 for abbreviations). Values in bold indicate significant *P* values after Bonferroni correction (n = 1000, *P* < 0.05).

| Sites |  | ECS | | | | | | |  | SCS | | | |  | JPS |
| --- | --- | --- | --- | --- | --- | --- | --- | --- | --- | --- | --- | --- | --- | --- | --- |
|  |  | JM | LYG | QD | ZS | WZ | XP | XM |  | MM | BH | DX | SY |  | FU |
| JM |  | — | 0.848 | 0.743 | 0.747 | 0.525 | 0.917 | 0.138 |  | **< 0.001** | **< 0.001** | **< 0.001** | **0.001** |  | **< 0.001** |
| LYG |  | - 0.055 | — | 0.443 | 0.440 | 0.473 | 0.682 | 0.196 |  | **< 0.001** | **< 0.001** | **< 0.001** | **< 0.001** |  | **< 0.001** |
| QD |  | - 0.020 | 0.002 | — | 0.606 | 0.268 | 0.533 | 0.005 |  | **< 0.001** | **< 0.001** | **< 0.001** | **< 0.001** |  | **< 0.001** |
| ZS |  | - 0.021 | 0.000 | - 0.008 | — | 0.550 | 0.226 | **< 0.001** |  | **< 0.001** | **< 0.001** | **< 0.001** | **< 0.001** |  | **< 0.001** |
| WZ |  | - 0.010 | - 0.001 | 0.020 | - 0.006 | — | 0.133 | 0.170 |  | **< 0.001** | **< 0.001** | **< 0.001** | **< 0.001** |  | **< 0.001** |
| XP |  | - 0.054 | - 0.029 | - 0.005 | 0.017 | 0.054 | — | 0.014 |  | **< 0.001** | **< 0.001** | **< 0.001** | **< 0.001** |  | **< 0.001** |
| XM |  | 0.125 | 0.115 | 0.144 | 0.187 | 0.145 | 0.137 | — |  | **< 0.001** | **< 0.001** | **< 0.001** | **< 0.001** |  | **0.001** |
| MM |  | 0.928 | 0.931 | 0.908 | 0.915 | 0.937 | 0.925 | 0.708 |  | — | 0.239 | 0.971 | 0.573 |  | **< 0.001** |
| BH |  | 0.938 | 0.941 | 0.916 | 0.922 | 0.948 | 0.934 | 0.709 |  | 0.026 | — | 0.051 | 0.403 |  | **< 0.001** |
| DX |  | 0.934 | 0.938 | 0.913 | 0.920 | 0.944 | 0.930 | 0.710 |  | - 0.077 | 0.096 | — | 0.416 |  | **< 0.001** |
| SY |  | 0.926 | 0.931 | 0.903 | 0.910 | 0.937 | 0.922 | 0.670 |  | 0.023 | 0.003 | 0.004 | — |  | **< 0.001** |
| FU |  | 0.933 | 0.938 | 0.898 | 0.901 | 0.949 | 0.921 | 0.656 |  | 0.887 | 0.908 | 0.897 | 0.893 |  | — |
